# High-quality draft genome sequences of *Acinetobacter pittii* strains isolated from soil in Gwagwalada, Nigeria

**DOI:** 10.1128/mra.00338-25

**Published:** 2025-06-09

**Authors:** Ahmed Olowo-okere, E. Skiebe, G. Wilharm

**Affiliations:** 1 Department of Pharmaceutical Microbiology and Biotechnology, Faculty of Pharmaceutical Sciences, University of Abuja 99399, Abuja, Federal Capital Territory, Nigeria; 2 Project Group P2, Robert Koch Institute 235859, Wernigerode, Germany; DOE Joint Genome Institute, Berkeley, California, USA

**Keywords:** *Acinetobacter pitti*, draft genome, soil, Nigeria, antibiotic resistance

## Abstract

We report the draft genome sequences of six *Acinetobacter pittii* strains isolated from soil in Gwagwalada, Nigeria. The genomes range in size from 3.73 to 3.95 Mb, GC contents of 38.84%–38.97%, and high completeness (99.99%–100%). Annotation identified 3,532-3,753 coding genes and multiple AMR determinants.

## ANNOUNCEMENT

The genus *Acinetobacter* comprises Gram-negative, non-fermentative bacteria widely distributed in natural and clinical environments (1, 2). *Acinetobacter pittii*, a member of the *Acinetobacter calcoaceticus-baumannii* complex, is an emerging opportunistic pathogen with increasing clinical relevance (3). Despite its significance, genomic data on environmental isolates of *A. pittii* remain highly limited. Here, we report the high-quality draft genome sequences of six *A. pittii* strains isolated from soil in Gwagwalada, Nigeria.

Approximately 30 g of soil samples was collected from a depth of about 20 cm using a sterile spatula. The description of the nature of soil collected and the geographical coordinates of the sampling points have been detailed in Table 1. About 1 g of the collected soil sample was pre-enriched in 10 mL of mineral salt medium (MM) supplemented with 0.2% sodium acetate for 5 h and subsequently plated on CHROMagar Acinetobacter (without multidrug-resistant supplement) and incubated overnight at 37°C as described previously (4). Colonies with morphological characteristics consistent with *Acinetobacter* species were selected and purified on Columbia agar supplement with 5% sheep blood.

**TABLE 1:** Assembly and genome annotation metrics

Feature	A27-3	A22-2-2	A27-1	B3_1	C1_3	B11_1
Accession number	Jbmdlk000000000	Jbmdlj000000000	Jbmdli000000000	Jbmdlh000000000	Jbmdlg000000000	Jbmdll000000000
Reads accession	SRR32962587	SRR32962586	SRR32962585	SRR32962584	SRR32962583	SRR32973066
Read counts	9869010	11154944	14430984	7637478	5727628	8154738
Average read length	246	260	248	277	270	247
Sampling points description	Turkey and rabbit sheds	Combined animal sheds (Cattle and goats)	Turkey and rabbit sheds	Grassland with diverse species	Uncultivated free-growing weed area within the University of Abuja	Termite mounds
Geographical coordinate	8°54'34"N 7°05'08"E	9°06'19"N 7°13'08"E	8°54'34"N 7°05'08"E	8°58'50"N 7°10'24"E	8°59'02"N 7°10'23"E	8°58'41"N 7°10'28"E
Completeness (%)	100	99.99	100	100	100	99.99
Contamination (%)	0.04	0.22	0.04	0.11	0.02	0.13
Genome size (bp)	3,854,265	3,945,058	3,874,361	3,736,982	3,949,930	3,912,971
GC content (%)	38.94	38.88	38.86	38.94	38.84	38.97
Contig N50 (bp)	45,846	48,269	74,982	44,627	114,305	58,338
Total contigs	154	145	105	171	74	127
Max contig length (bp)	201,862	175,091	301,772	168,521	457,155	142,742
Total coding sequences	3,662	3,751	3,667	3,532	3,753	3,713
RNA genes	55	60	58	57	70	65
ANI (%)	96.5	96.6	96.5	96.6	96.5	96.5
Predicted AMR genes	*abeS, adeG, adeF, adeH, ADC-21, AbaQ, adeK, adeJ, adeI, OXA-417, abeM, AmvA*	*abeM, abeS, adeL, adeG, adeF, adeH, ADC-15, AmvA, OXA-417, adeK, adeJ, adeI*	*abeM, abeS, adeG, adeF, adeH, ADC-21, AbaQ, adeK, adeJ, adeI, OXA-417, AmvA*	*ADC-43, adeH, adeF, adeG, adeL, abeS, abeM, adeK, adeJ, adeI, OXA-417, AbaQ, AmvA*	*abeM, ADC-22, adeH, adeF, adeG, adeL, abeS, AmvA, AbaF, OXA-417, adeI, adeJ, adeK, AbaQ*	*abeM, ADC-41, adeH, adeF, adeG, adeL, abeS, adeK, adeJ, adeI, OXA-417, AbaQ, AbaF, AmvA*

Genomic DNA from an overnight culture of the strain in Luria-Bertani (LB) broth was extracted using MasterPure DNA purification kit (Epicentre) and quantified using the Invitrogen Qubit Assay. Sequencing was performed on an Illumina NextSeq platform (2 × 300 bp) using Nextera XT DNA Library Prep Kit according to the manufacturer’s instruction. The genome sequence was *de novo* assembled using the SPAdes v4.0 algorithm implemented through Shovill v1.1.0 (5, 6). The completeness, contamination, and genomic metrics were assessed using CheckM2 v1.0.2 and QUAST v5.2.0 (7, 8). The genome functional annotation was performed using the NCBI Prokaryotic Genome Annotation Pipeline (9). The taxonomic position of the strain was confirmed through pairwise average nucleotide identity (ANI) comparison as determined by fastANI v1.33 against the type strain of *A. pittii* FDAARGOS 1399^T^ (ASM1904720v1) (10). Antibiotic resistance genes were later identified using the NCBI AMRFinder v4.0.19 (11)

Sequencing generated between 5727628 and 14430984 paired-end reads. The genomes of the six *A. pittii* strains (ABJ_A27_3, ABJ_A22_2_2, ABJ_A27_1, ABJ_B3_1, ABJ_C1_3, and ABJ_B11_1) range in size from 3.73 to 3.95 Mb, with GC contents of 38.84%–38.97%. The assemblies consist of 74–171 contigs, with N50 values ranging from 44,627–114,305 bp. Completeness estimates were 99.99%–100%, with low contamination levels (0.02%–0.22%). Genome annotation identified 3,532–3,753 protein-coding genes, along with 55–70 RNA genes (Table 1). Pairwise ANI comparison revealed that the genomes of the six strains had >95% (96.5%–96.6%) nucleotide identity with the *A. pittii* FDAARGOS 1399^T^ (ASM1904720v1) (Table 1).

Multiple AMR genes, including efflux pumps (*adeFGH*, *adeIJK*, *AbaQ*, *AbaF*, *AmvA*), β-lactamases (*OXA-417*, *ADC variants*), and multidrug transporters (*abeM*, *abeS*) were detected in the genomes of the six strains. These genes were predicted to confer resistance to various antibiotics (Table 1).

The draft genome sequences of these *A. pittii* strains provide valuable insights into their genomic features and potential ecological and clinical significance.

## Data Availability

The draft genomes of the six A. pitti strains have been deposited at DDBJ/ENA/GenBank under BioProject accession PRJNA1183826. Genome accessions are available in Table 1.
